# Causal Relationship Between Gut Microbiota and Gastrointestinal Polyps: A Mendelian Randomization Study

**DOI:** 10.5152/tjg.2025.24347

**Published:** 2025-01-06

**Authors:** Yang Xie, Sheng Chen, Yiling Xiong, Chunyan Zeng, Youxiang Chen

**Affiliations:** 1 Department of Gastroenterology, Jiangxi Provincial Key Laboratory of Digestive Diseases, Jiangxi Clinical Research Center for Gastroenterology, Digestive Disease Hospital, The First Affiliated Hospital, Jiangxi Medical College, Nanchang University, Jiangxi, China; 2 Department of Gastroenterology, Jiangxi Province Hospital of Integrated Chinese and Western Medicine, Jiangxi, China

**Keywords:** Gut microbiota, Mendelian randomization, gastrointestinal polyps

## Abstract

**Background/Aims:**

Numerous studies have confirmed that intestinal flora is closely linked to the development of gastrointestinal polyps. However, the precise causal link between them has yet to be clarified. This study sought to determine the causal relationship between gut microbiota and gastric, duodenal, colon, and rectal polyps by Mendelian randomization (MR).

**Materials and Methods:**

We employed publicly available genome-wide association study summary data to conduct MR analysis. Gut microbiota data were sourced from the International MiBioGen Consortium, and gastrointestinal polyp data were obtained from the MRC-IEU Consortium. Instrumental variables were selected based on eligible single-nucleotide polymorphisms. To assess causality, we utilized MR-Egger, weighted median, inverse variance weighting, simple mode, and weighted mode techniques. Heterogeneity and pleiotropy were evaluated through Cochran’s Q test, MR-Egger intercept test, and leave-one-out analysis.

**Results:**

We determined that Lachnospiraceae UCG004, Erysipelotrichaceae UCG003, and Veillonella increased the risk of colon polyps. However, Dorea and *Clostridium innocuum* group act as protective factors for colon polyps. Allisonella increases the risk of rectal polyps. In contrast, Christensenellaceae R.7 group, Parasutterella, and Intestinimonas are protective factors for rectal polyps. Lachnospiraceae FCS020 group, Intestinibacter, Ruminococcaceae UCG003, and Parasutterella act as risk factors for stomach and duodenum polyps.

**Conclusions:**

Our research establishes a causal link between gut microbiota dysbiosis and the formation of gastrointestinal polyps. Nonetheless, additional studies are necessary to explore the mechanisms through which bacterial taxa influence the development of these polyps.

**Main Points**

- The study provides strong evidence confirming a causal relationship between gut microbiota dysbiosis and the development of gastrointestinal polyps, specifically highlighting that certain bacterial taxa can either increase or decrease the risk of polyps in different parts of the gastrointestinal tract.
- Key bacterial groups were identified as either risk factors or protective factors for various types of polyps. For instance, Lachnospiraceae UCG004, Erysipelotrichaceae UCG003, and Veillonella were associated with an increased risk of colon polyps, while Dorea and *Clostridium innocuum* group were found to be protective.
- Future research and therapeutic targets: The findings suggest that further investigation into the mechanisms by which specific bacteria influence polyp development could lead to the discovery of new therapeutic targets. These insights have the potential to guide the prevention and treatment of gastrointestinal polyps and associated neoplastic progression.

## Introduction

Gastrointestinal polyps are elevated lesions of the mucosa of the gastrointestinal tract that protrude into the lumen and can be divided into inflammatory polyps, adenomatous polyps, dysplastic polyps, and hyperplastic polyps.^1^ Adenomatous polyps progress to colorectal cancer and are the main component of malignant polyps.^2^ Apart from regular gastroenteroscopy and resection, there are no effective therapeutic drugs, and the recurrence rate of intestinal polyps has been reported to be 20%-50%.^3^ Early detection of precancerous polyps and early removal of precancerous lesions are essential to reduce morbidity and mortality in GI cancers.^4^ Early detection and removal of pre-cancerous lesions can reduce cancer morbidity and mortality by about 50%.^5^

The human microbiota is highly diverse and can exert both positive and negative effects on health.^6^ Multiple studies have suggested a possible causal connection between alterations in the gut microbiota and the emergence of gastrointestinal polyps.^7^ These observations have prompted us to hypothesize that the intestinal microbiota might be significantly related to the development of intestinal polyps. However, different regions of the gastrointestinal tract harbor distinct microorganisms. Therefore, the true causal link between gut microbiota and gastrointestinal polyps is unclear and needs to be further elucidated.^8^

Mendelian randomization (MR) uses genetic variants closely associated with exposure as instrumental variables (IVs) to derive causal linkages between risk variables and health outcomes.^9^ Unlike observational studies, which are susceptible to confounding effects, reverse causality, and other biases, MR provides a more robust approach, overcoming these limitations to yield reliable results.^10^ Mendelian randomization has emerged as a powerful method for investigating questions in human biology and epidemiology, including the association between the gut microbiota and disease.^11^ Notably, prior investigations have examined the causal effect of gut microbiota in the formation of gastrointestinal polyps using MR analysis. Our study utilized an extensive dataset of polyps located in three different sites: gastric and duodenal polyps, colonic polyps, and rectal polyps. The causal relationship between the gut microbiota and the development of gastrointestinal polyps was investigated by two-sample MR analyses. This work provides new theoretical and empirical evidence for the prevention and treatment of gastrointestinal polyps.

## Materials and Methods

### Research Design

**Figure 1.**
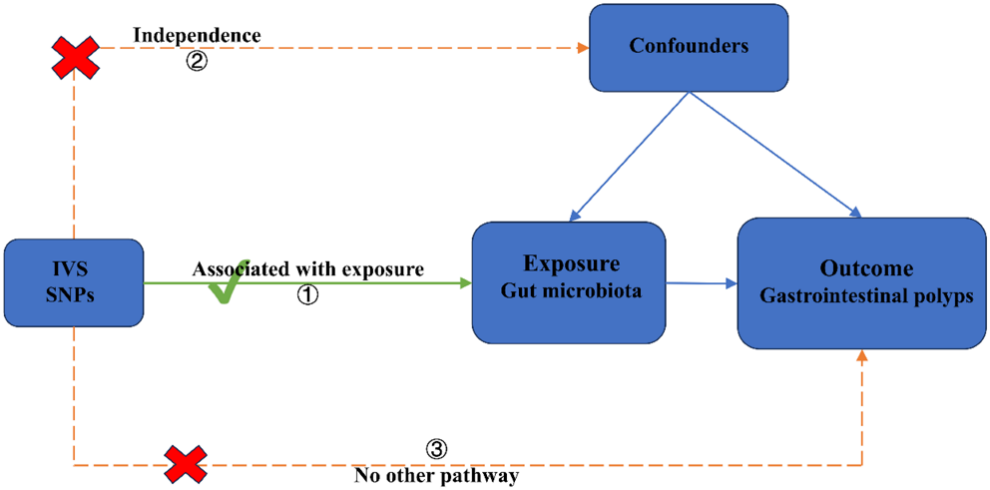
Schematic overview of the study design.

We performed a two-sample MR study to investigate the causal role of gut microbiota on gastrointestinal polyps (Figure 1). This MR method is based on 3 key assumptions: (1) the IV derived from genetic variation is strongly related to gut microbiota; (2) genetic variation is not associated with confounders; and (3) genetic variation affects pneumonia risk only by affecting the gut microbiota and does not involve any other pathway. Our analysis primarily relies on evidence from independent genome-wide association study (GWAS).

## Data Sources

### Exposure Data Sources—Gut Microbiota

The MiBioGen consortium is an international collaboration focused on understanding the genetic structure of the gut microbiota. This group has compiled data from 24 population-based cohorts totaling 18 340 individuals. Within each cohort, the gut microbiota was analyzed using 16S rRNA sequencing, while participants were genotyped with extensive single-nucleotide polymorphism (SNP) arrays.^12^ Genotype imputation was performed using the HRC 1.0 or 1.1 reference panels. In this study, 131 genera were identified, with 119 considered as exposures, excluding 12 unknown genera.^13^

### Outcome Data Sources—Gastrointestinal Polyp

The combined GWAS data set for gastrointestinal polyps was derived from the most extensive recent studies, involving cases and controls from the UK Biobank (Colon polyp: ukb-b-1968; Rectal polyp: ukb-b-19805; Stomach and duodenum polyp: ukb-b-7330). Colon polyp dataset included 463 010 individuals of European ancestry, comprising 4779 patient cases and 458 231 controls. The rectal polyp genome study included 463 010 individuals of European ancestry, with 2800 patients and 460 210 controls. In addition, the dataset for the stomach and duodenum polyp group comprised 430 010 individuals of European descent, including 1053 patients and 461 957 controls. These datasets were sourced from previously published studies and did not necessitate separate ethical approval.

### Genetic Instrumental Variables

To ascertain the precision and reliability of the study outcomes, the following quality control routines were employed in the selection of appropriate genetic IVs: (1) significance threshold: *P *< 1.0 × 10^−5^.^14^ (2) Clumping process: *R*^2^ < 0.001 within a 10 000 kb window, reducing linkage disequilibrium (LD) and ensuring random assortment during gestation. (3) Exclusion of palindromic SNPs: alleles that were incompatible or had intermediate allele frequencies were excluded. (4) Instrument strength: F-statistics (BETA^2^/SE^2^) for each SNP were calculated, excluding weak IVs with *F* < 10.^15^ The IVs of this study are summarized in Supplementary Table 1.

### Statistical Analysis

In the primary analysis, we used inverse variance weighting (IVW) meta-analysis to generate estimates. This method combines the Wald values for each SNP and derives overall effect estimates through meta-analysis techniques.^16^ If heterogeneity is detected among the SNPs in the analysis, we will apply the random-effects IVW method.^17^ All statistical analyses in our investigation, encompassing both MR and sensitivity analyses, were executed using the R packages “TwoSampleMR” and “MRPRESSO” within the publicly available R software version 4.3.3 (R Foundation for Statistical Computing, Vienna, Austria).

### Sensitivity Analyses

To ensure the reliability of the genetic causal effects, we utilized several methods: MR-Egger, simple mode, weighted median, and weighted mode, all of which provide robust evidence under varying conditions.^18^ PhenoScanner database (http://www.phenoscanner.medschl.cam.ac.uk/) was queried to check if the selected SNPs were linked to potential confounding traits (e.g., BMI) at a significance threshold of 1 × 10^−5^. Data were reanalyzed after removing these SNPs. Additionally, Cochran’s *Q* statistic test was used to assess heterogeneity, and sensitivity analysis methods such as “leave-one-out,” forest plots, scatter plots, and funnel plots were used to visualize the sensitivity of our findings.

## Results

Supplementary Table 1 lists the characteristics of the selected SNPs for each gut microbiota along with the variance values and F-statistics.

### The Effect of Gut Microbiota on Colon Polyp

**Figure 2.**
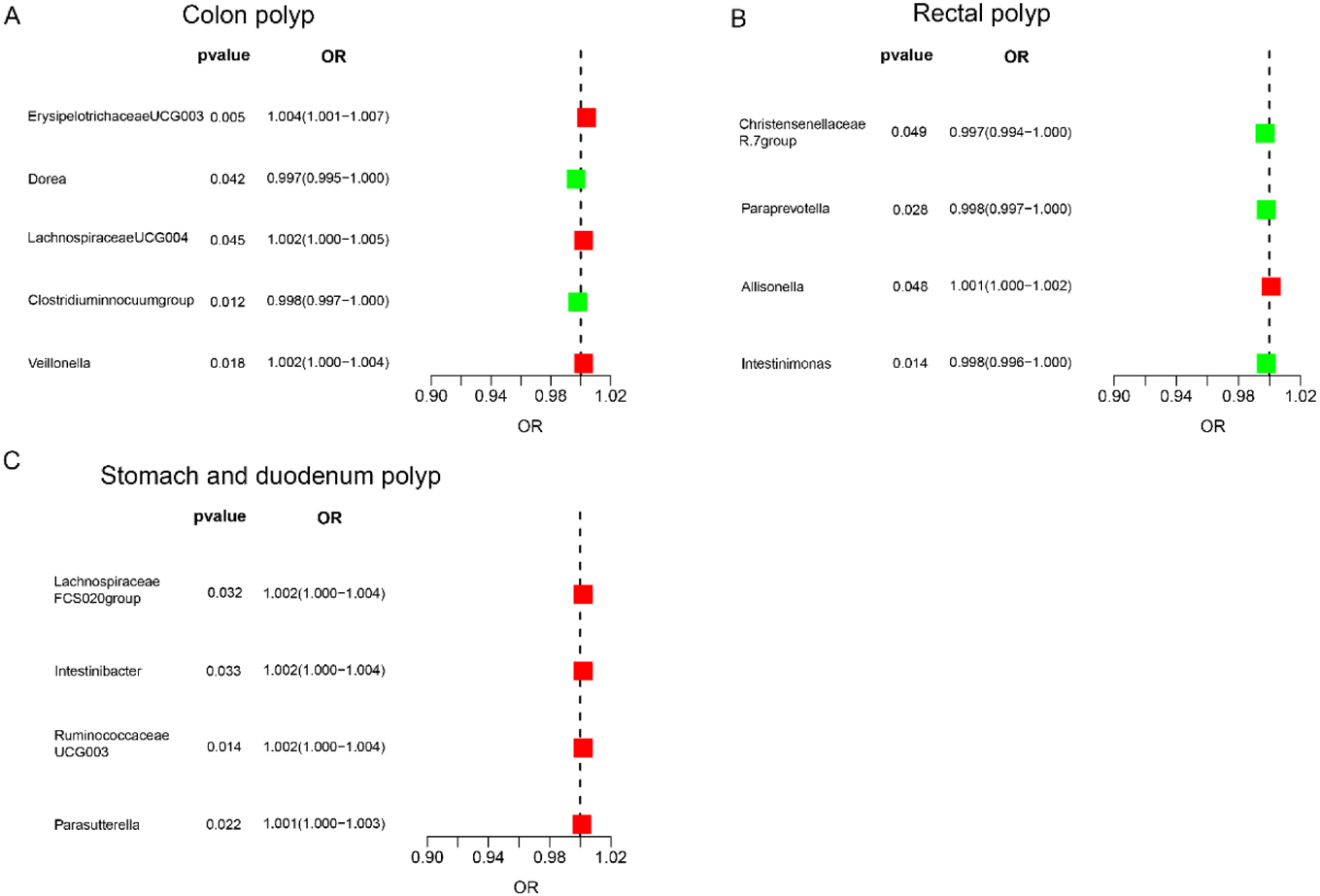
Forest plot of associations between genetically determined gut microbiota and gastrointestinal polyps. (A) Colon polyp, (B) rectal polyp, and (C) stomach and duodenum polyp.

The IVW analysis revealed the following associations in the colon region: Erysipelotrichaceae UCG003 (OR = 1.004, 95% CI: 1.001-1.007), Lachnospiraceae UCG004 (OR = 1.002, 95% CI: 1.000-1.005), and Veillonella (OR = 1.002, 95% CI: 1.000-1.004) were positively correlated with colon polyp risk (Figure 2A, Table 1). Conversely, *Clostridium innocuum* group (OR = 0.998, 95% CI: 0.997-1.000) and Dorea (OR = 0.997, 95% CI: 0.995-1.000) were suggested to have a protective effect against colon polyps (Figure 2A, Table 1).

### The Effect of Gut Microbiota on Rectal Polyp

In the rectal region, Allisonella (OR = 1.001, 95% CI: 1.000-1.002) was positively correlated with rectal polyp risk (Figure 2B, Table 1). Conversely, Christensenellaceae R.7 group (OR = 0.997, 95% CI: 0.994-1.000), Intestinimonas (OR=0.998, 95% CI: 0.996-1.000), and Paraprevotella (OR = 0.998, 95% CI: 0.997-1.000) were suggested to have a protective effect against rectal polyps (Figure 2B, Table 1).

### The Effect of Gut Microbiota on Stomach and Duodenum Polyp

Finally, in the stomach and duodenum region, Lachnospiraceae FCS020 group (OR = 1.002, 95% CI: 1.000-1.004), Intestinibacter (OR = 1.002, 95% CI: 1.000-1.004), Ruminococcaceae UCG003 (OR = 1.002, 95% CI: 1.000-1.004), and Parasutterella (OR = 1.001, 95% CI: 1.000-1.003) were positively correlated with the risk of stomach and duodenum polyps (Figure 2C, Table 1). Detailed MR results can be found in Supplementary Table 2.

### Sensitivity Analysis

**Figure 3.**
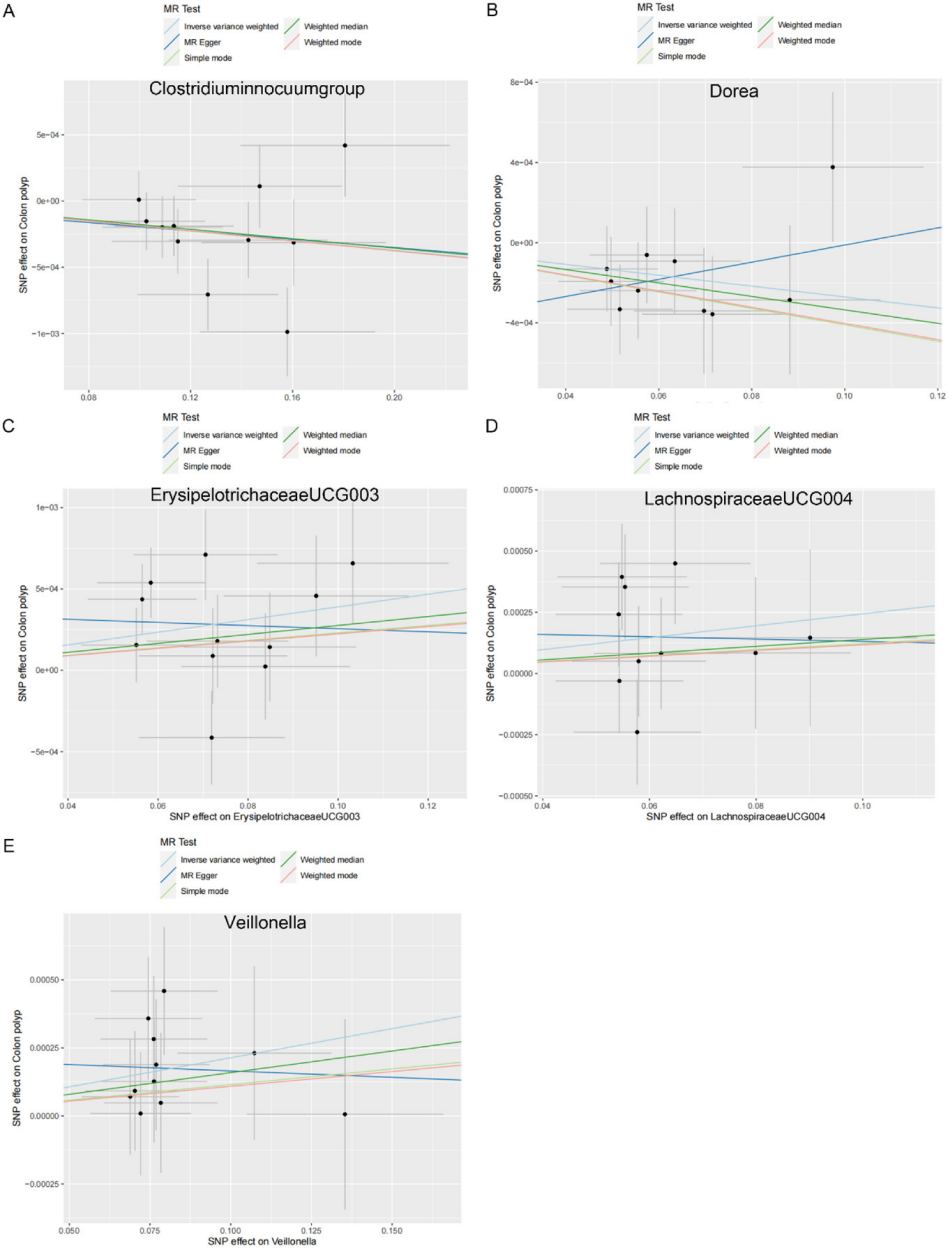
Scatter plots showing the causal effect of SNPs on gut microbiota versus colon polyp. (A) *Clostridium innocuum* group; (B) Dorea; (C) Erysipelotrichaceae UCG003; (D) Lachnospiraceae UCG004; and (E) Veillonella. MR, Mendelian randomization; SNP, single-nucleotide polymorphisms.

**Figure 4.**
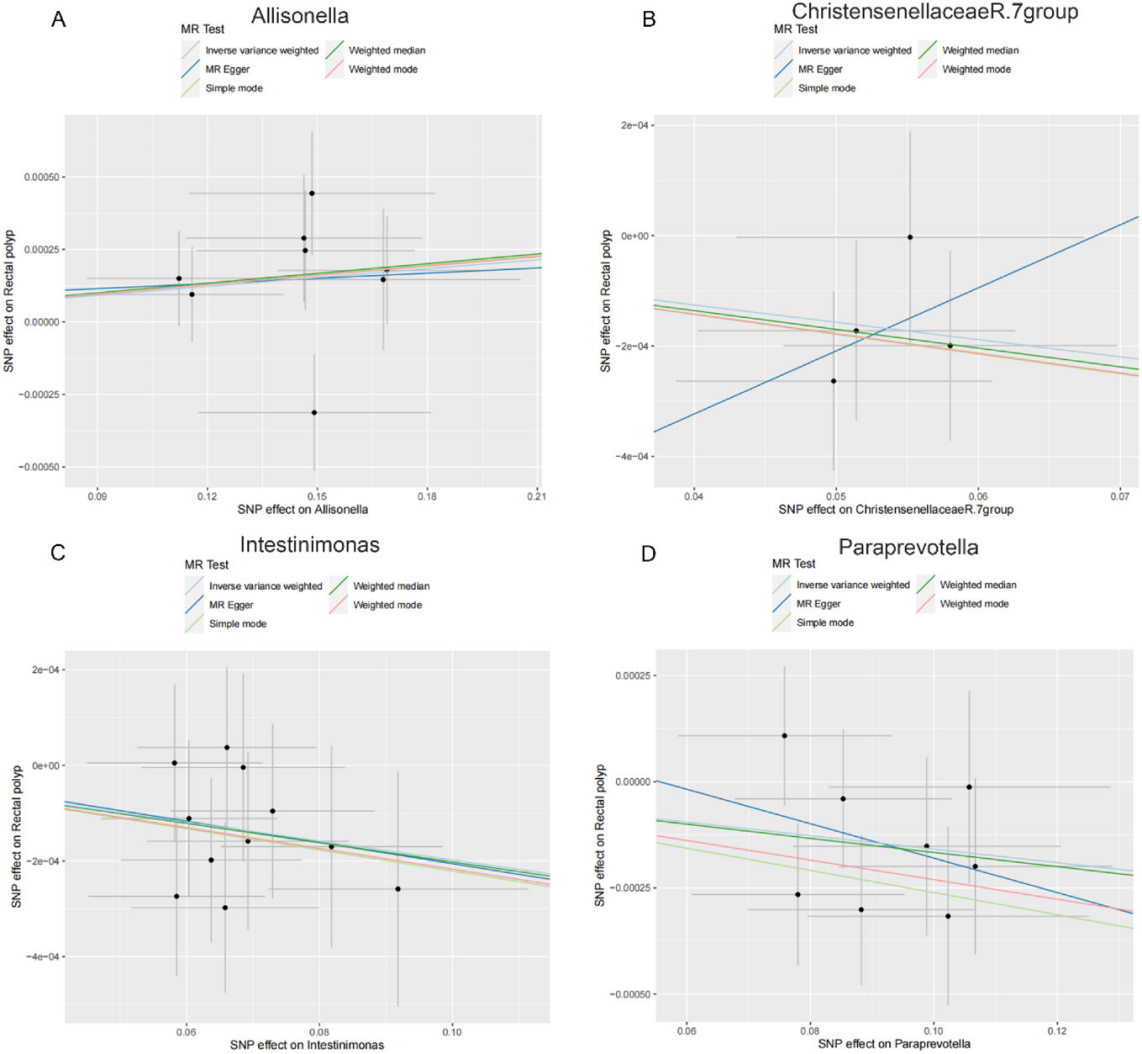
Scatter plots showing the causal effect of SNPs on gut microbiota versus rectal polyp. (A) Allisonella; (B) Christensenellaceae R.7 group; (C) Intestinimonas; and (D) Paraprevotella. MR, Mendelian randomization; SNP, single-nucleotide polymorphisms.

**Figure 5.**
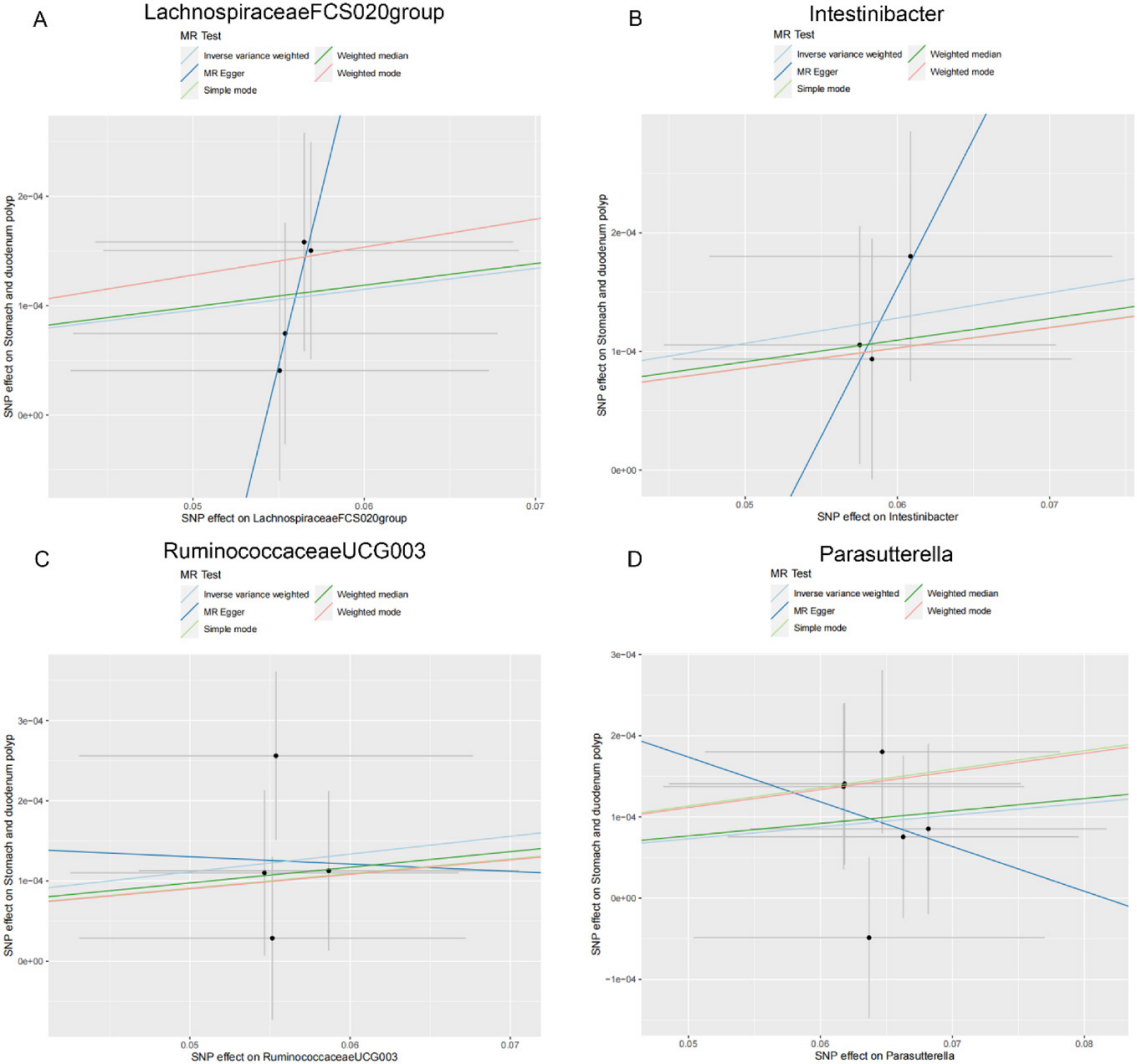
Scatter plots showing the causal effect of SNPs on gut microbiota versus stomach and duodenum polyp. (A) Lachnospiraceae FCS020group; (B) Intestinibacter; (C) Ruminococcaceae UCG003; and (D) Parasutterella. MR, Mendelian randomization; SNP, single-nucleotide polymorphisms.

**Supplementary Figure 1.**
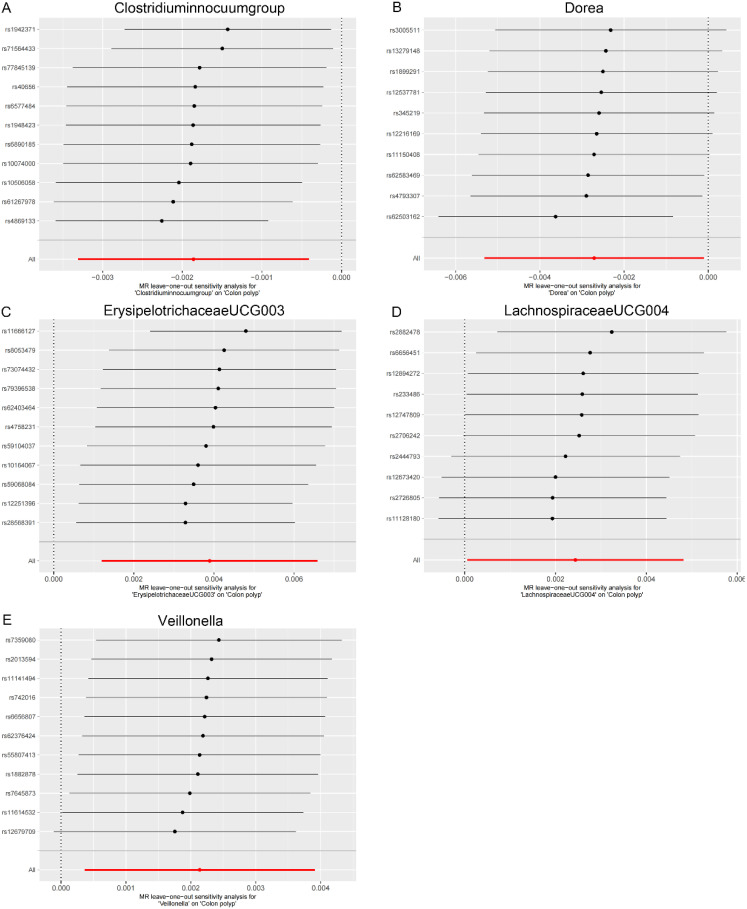
Leave-one-out sensitivity analysis based on the IVW model for the association between gut microbiota and colon polyp. (A) *Clostridium innocuum* group; (B) Dorea; (C) Erysipelotrichaceae UCG003; (D) Lachnospiraceae UCG004; (E) Veillonella. The red horizontal line represents the overall estimate, while the black horizontal line represents the estimate after removing a single variant. Abbreviations: SNP, single-nucleotide polymorphism; IVW, Inverse variance weighted.

**Supplementary Figure 2.**
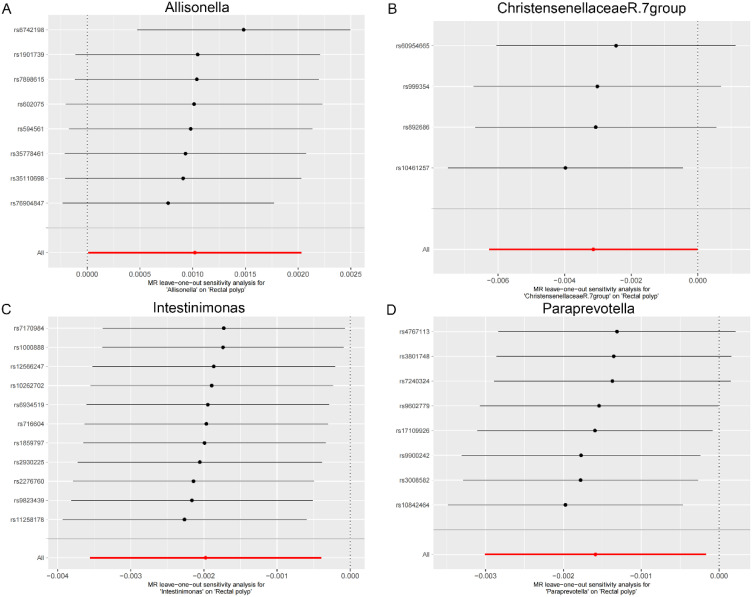
Leave-one-out sensitivity analysis based on the IVW model for the association between gut microbiota and rectal polyp. (A) Allisonella; (B) Christensenellaceae R.7 group; (C) Intestinimonas; (D) Paraprevotella. The red horizontal line represents the overall estimate, while the black horizontal line represents the estimate after removing a single variant. Abbreviations: SNP, single-nucleotide polymorphism; IVW, Inverse variance weighted.

**Supplementary Figure 3.**
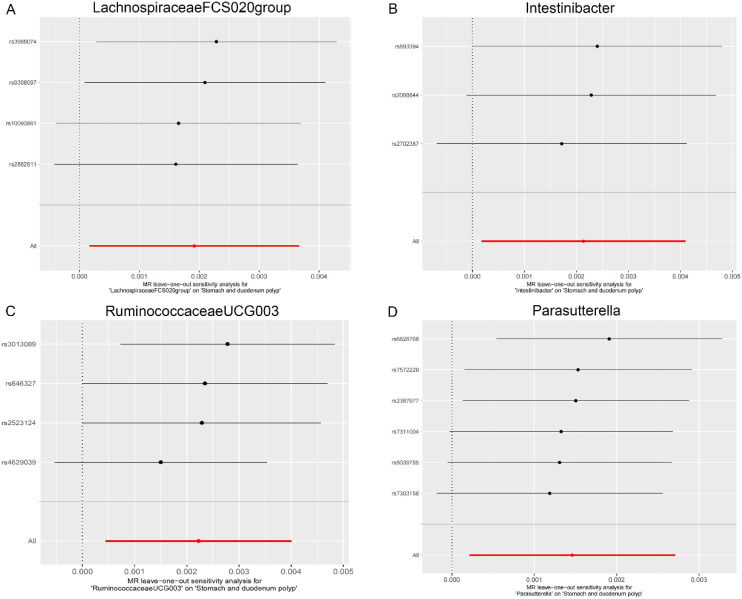
Leave-one-out sensitivity analysis based on the IVW model for the association between gut microbiota and stomach and duodenum polyp. (A) Lachnospiraceae FCS020 group; (B) Intestinibacter; (C) Ruminococcaceae UCG003; (D) Parasutterella. The red horizontal line represents the overall estimate, while the black horizontal line represents the estimate after removing a single variant. Abbreviations: SNP, single-nucleotide polymorphism; IVW, Inverse variance weighted.

**Supplementary Figure 4.**
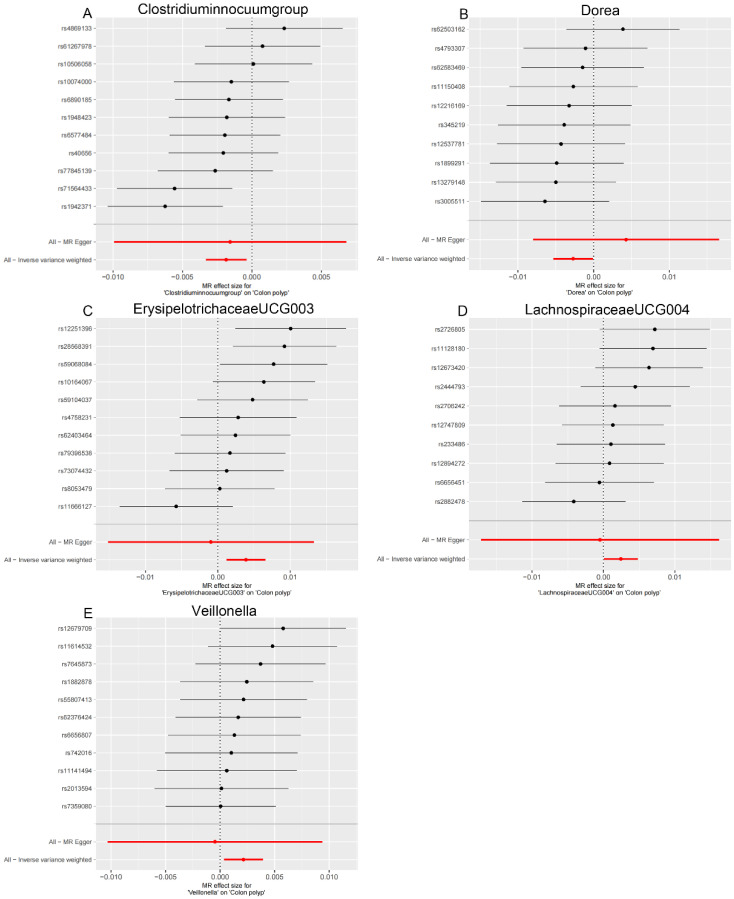
Forest plot for the association between Gut microbiota on colon polyp. (A) *Clostridium innocuum* group; (B) Dorea; (C) Erysipelotrichaceae UCG003; (D) Lachnospiraceae UCG004; (E) Veillonella. Inverse Variance Weighting (IVW) and MR Egger methods were used to detect the heterogeneity of SNP.

**Supplementary Figure 5.**
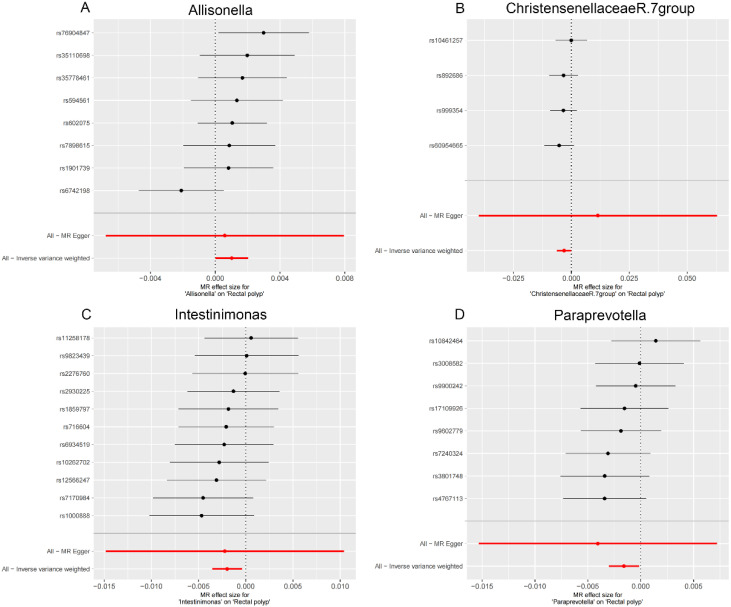
Forest plot for the association between Gut microbiota on rectal polyp. (A) Allisonella; (B) Christensenellaceae R.7 group; (C) Intestinimonas; (D) Paraprevotella. Inverse Variance Weighting (IVW) and MR Egger methods were used to detect the heterogeneity of SNP.

**Supplementary Figure 6.**
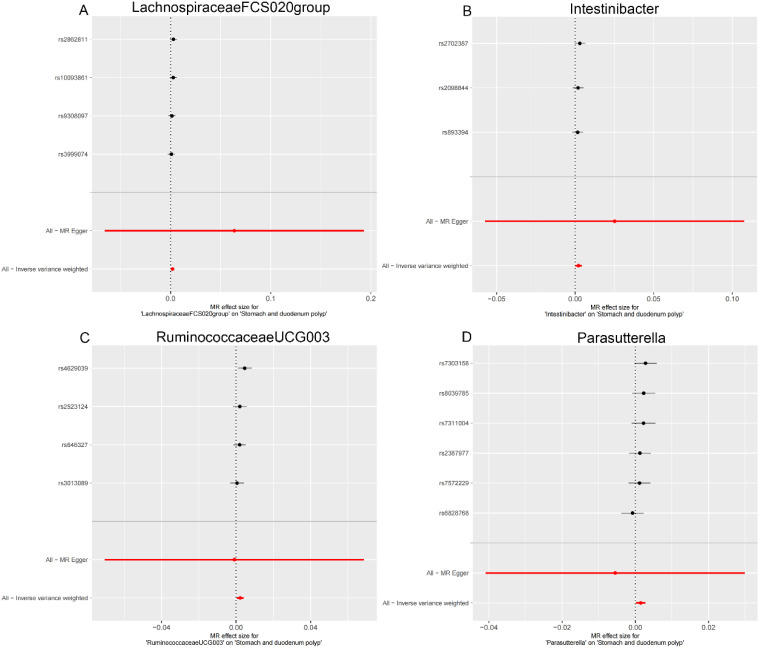
Forest plot for the association between Gut microbiota on stomach and duodenum polyp. (A) Lachnospiraceae FCS020 group; (B) Intestinibacter; (C) Ruminococcaceae UCG003; (D) Parasutterella. Inverse Variance Weighting (IVW) and MR Egger methods were used to detect the heterogeneity of SNP.

**Supplementary Figure 7.**
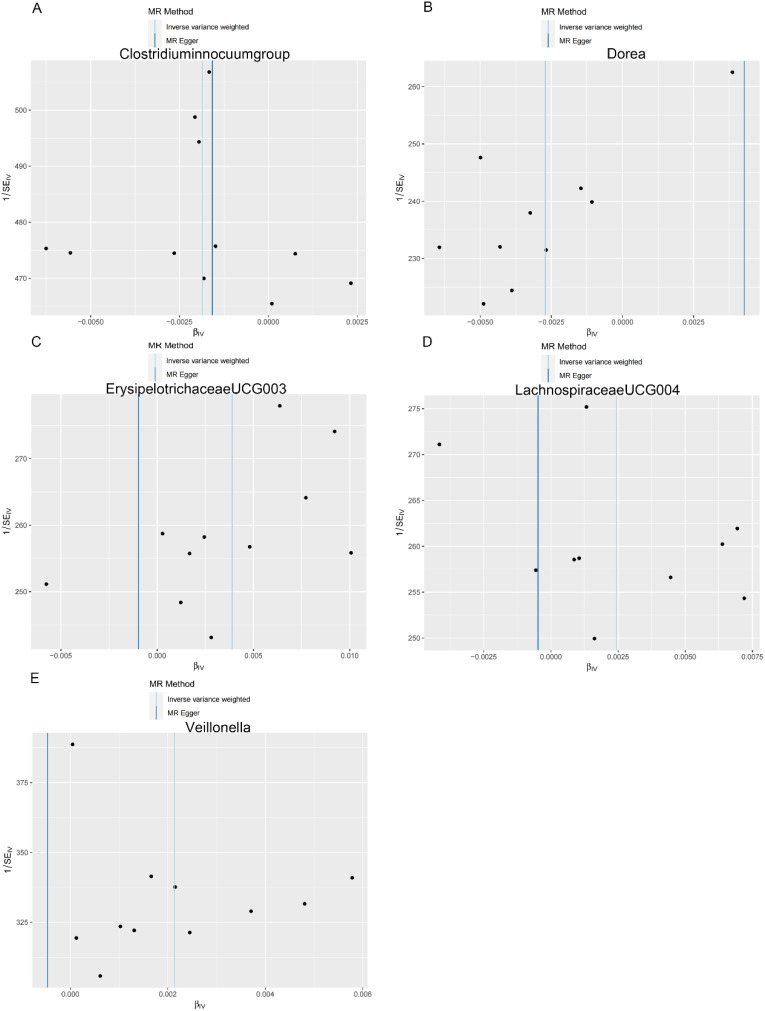
Funnel plots for MR analyses of the causal effect of Gut microbiota on colon polyp. (A) *Clostridium innocuum* group; (B) Dorea; (C) Erysipelotrichaceae UCG003; (D) Lachnospiraceae UCG004; (E) Veillonella. Inverse Variance Weighting (IVW) and MR Egger methods were used to detect the heterogeneity of SNP.

**Supplementary Figure 8.**
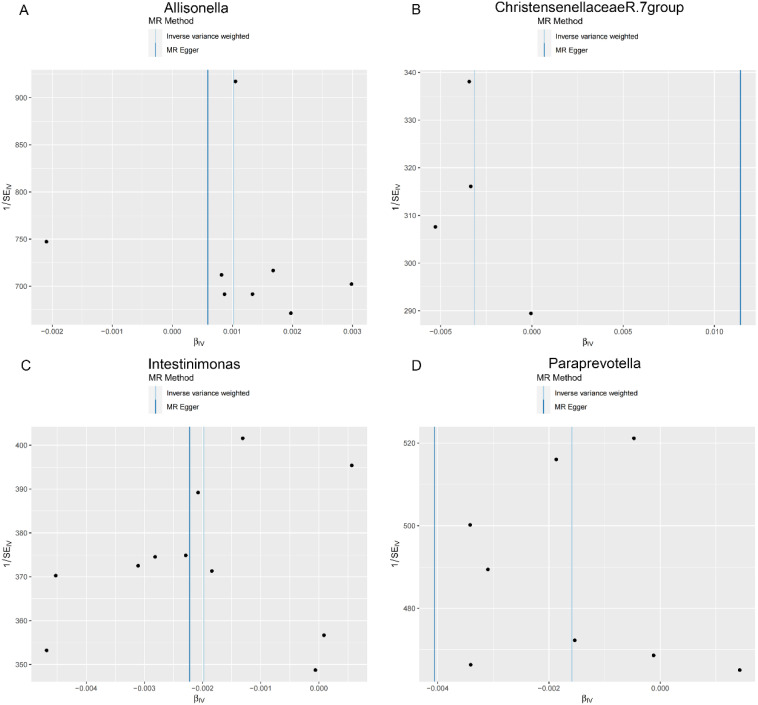
Funnel plots for MR analyses of the causal effect of Gut microbiota on rectal polyp. (A) Allisonella; (B) Christensenellaceae R.7 group; (C) Intestinimonas; (D) Paraprevotella. Inverse Variance Weighting (IVW) and MR Egger methods were used to detect the heterogeneity of SNP.

**Supplementary Figure 9.**
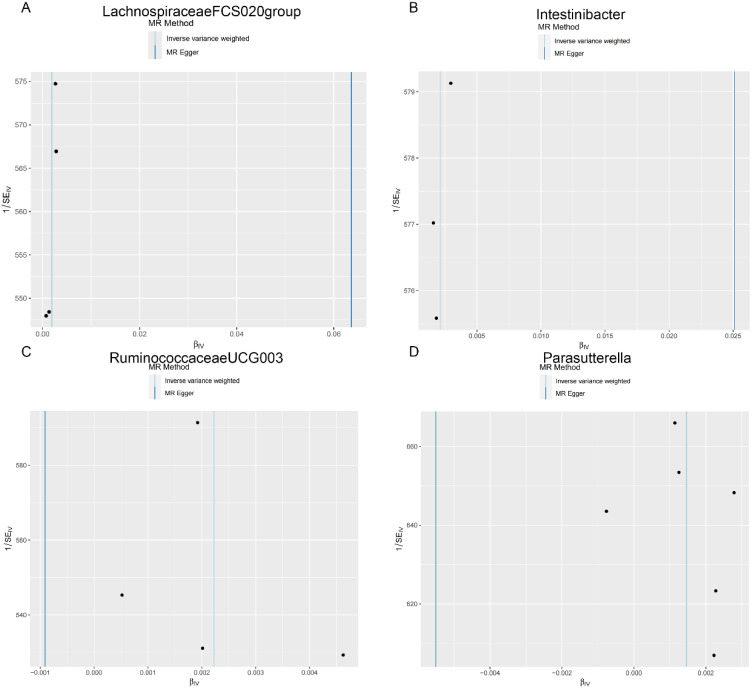
Funnel plots for MR analyses of the causal effect of Gut microbiota on stomach and duodenum polyp. (A) Lachnospiraceae FCS020 group; (B) Intestinibacter; (C) Ruminococcaceae UCG003; (D) Parasutterella. Inverse Variance Weighting (IVW) and MR Egger methods were used to detect the heterogeneity of SNP.

As shown in Table 2, both the IVW method and MR-Egger’s Cochran *Q *statistic reveal minimal heterogeneity and high reliability across these SNPs. Furthermore, the MR-PRESSO global test indicates no significant outliers affecting our estimation, with detailed results available in Supplementary Tables 3 and 4. Scatter plots illustrate the estimated effect sizes of SNPs within the gut microbiota in relation to gastrointestinal polyps (Figures 3-5). Moreover, a leave-one-out sensitivity analysis was performed using the IVW method, which showed consistent results even when individual SNPs were excluded, suggesting that any individual SNP did not unduly influence the overall estimate (Supplementary Figures 1-3). Forest plots are provided in Supplementary Figures 4-6. Additionally, funnel plots demonstrate overall symmetry, indicating little evidence of heterogeneity (Supplementary Figures 7-9).

## Discussion

In our MR investigation, we utilized the MiBioGen database and UK Biobank data to investigate the causal link between gut microbes and gastrointestinal polyps. Erysipelotrichaceae UCG003, Lachnospiraceae UCG004, and Veillonella were found to increase the risk of colon polyps, while Clostridium innocuum group and Dorea exhibited protective effects. For rectal polyps, Allisonella was associated with increased risk, whereas Christensenellaceae R.7 group, Intestinimonas, and Paraprevotella showed protective effects. Additionally, Lachnospiraceae FCS020 group, Intestinibacter, Ruminococcaceae UCG003, and Parasutterella were linked to an increased risk of stomach and duodenum polyps.

The human gut hosts a vast number of bacteria that have co-evolved with their human host, playing a crucial role in our physiology and metabolism.^19^ It is estimated that over 99% of these microorganisms are anaerobic, with only a small fraction being aerobic or mixed anaerobic bacteria. These gut bacteria have been linked to gastroenterology diseases.^20^ This study investigates the differences in microbiota between individuals with gastrointestinal polyps, focusing on different gut regions. Previous studies have revealed key associations between gastrointestinal polyps and Solobacterium moorei, suggesting that gut flora could serve as a non-invasive screening tool for intestinal polyps.^3^ Gut flora may contribute to the development of these polyps. In particular, Lachnospiraceae and Fusobacterium were identified more frequently in patients with gastrointestinal polyps compared to healthy controls.^21^ Additionally, the composition and diversity of salivary and fecal microbiota were found to differ significantly from those in healthy populations. These potential biomarkers show promise as non-invasive tools for detecting gastrointestinal polyps.^22^ Intestinal bacterial overgrowth indicates dysbiosis of the gut flora, which is linked to the pathogenesis of gastrointestinal polyps. The lactulose breath test was used to diagnose bacterial overgrowth in the small intestine and was a diagnostic tool for detecting intestinal dysbiosis, potentially preventing intestinal polyps by regulating intestinal flora.^23^ Bowel bacteria may promote the early stages of colorectal cancer through the development of adenomatous polyps.^24^ This study suggests that different types of intestinal polyps are associated with distinct intestinal flora, paving the way for targeted polyp prevention therapies. A previous study demonstrated that administering Indomethacin to mice induces shifts in the luminal microbiota, highlighting the impact of bacterial interactions on drug metabolism.^25^ This causal relationship is further supported by subsequent experiments showing that Celecoxib, a selective Cox-2 inhibitor, reduces the formation of precancerous adenomatous polyps in the gastrointestinal tract of humans and mice. This effect is achieved by modulating the intestinal flora, including a decrease in Lactobacillaceae and Bifidobacteriaceae, and an increase in Coriobacteriaceae.^26^

Although colorectal polyps are not cancerous, they can develop into colorectal cancer over time.^27^ Gastrointestinal polyps are typically detected and removed via gastroenteroscopy. However, many post-polypectomy patients are reluctant to undergo regular follow-up gastroenteroscopies.^28^ Testing for intestinal flora can increase patient compliance and provide an effective method for early screening of gastrointestinal polyps.

Our study has several advantages, notably the identification of associations between gastrointestinal polyps in different regions and specific types of intestinal flora. Erysipelotrichaceae UCG003, Lachnospiraceae UCG004, and Veillonella were found to increase the risk of colon polyps, while Allisonella was related to an increased risk of rectal polyps. Conversely, Christensenellaceae R.7 group, Lachnospiraceae FCS020 group, Intestinibacter, Ruminococcaceae UCG003, and Parasutterella were correlated with an increased risk of stomach and duodenum polyps. By indicating the location of intestinal polyps based on the abundance of different intestinal flora, we can potentially reduce the cost of gastroenteroscopy and improve the detection rate of polyps during the procedure. Identifying intestinal flora causally associated with gastrointestinal polyps offers a valuable new strategy for the prevention and treatment of these polyps mediated by the gut microbiota.

Nevertheless, this study has limitations. All gastrointestinal polyp’s data were sourced from individuals of European ancestry, while the gut flora database includes data from other populations. Therefore, our results are not necessarily applicable to other ethnicities.^29^ Furthermore, although our findings identified a causal relationship between intestinal flora and gastrointestinal polyps, the precise mechanisms by which these gut microbes influence the development of polyps remain unclear. Therefore, further studies are needed in the future to elucidate the mechanistic influence of gut microbes on the development of intestinal polyps.

In conclusion, we identified a causal relationship between gut microbiota dysbiosis and gastrointestinal polypogenesis by MR analysis. Further randomized controlled trials are needed to elucidate the mechanisms underlying the etiology of gastrointestinal polyps due to specific bacterial taxa.

### Acknowledgments:

The authors would also want to acknowledge all the previous studies and databases that were devoted to data collation, analysis, summary, and public accessibility.

## Supplementary Materials

Supplementary Material

## Figures and Tables

**Table 1.** MR Estimates for the Association Between Gut Microbiota and Gastrointestinal Polyps

Outcomes	Exposures	N SNP	MR Method	OR (95% CI)	*P*
Colon polyp	*Clostridium innocuum* group	11	MR Egger	0.998 (0.990-1.007)	.720
			Weighted median	0.998 (0.996-1.000)	.052
			IVW	0.998 (0.997-1.000)	**.012**
			Simple mode	0.998 (0.995-1.001)	.202
			Weighted mode	0.998 (0.995-1.001)	.196
	Dorea	10	MR Egger	1.004 (0.992-1.017)	.513
			Weighted median	0.997 (0.993-1.000)	.050
			IVW	0.997 (0.995-1.000)	**.042**
			Simple mode	0.996 (0.990-1.001)	.181
			Weighted mode	0.996 (0.990-1.001)	.196
	Erysipelotrichaceae UCG003	11	MR Egger	1.000 (0.985-1.013)	.897
			Weighted median	1.003 (0.999-1.006)	.111
			IVW	1.004 (1.001-1.007)	**.005**
			Simple mode	1.002 (0.997-1.008)	.439
			Weighted mode	1.002 (0.997-1.007)	.418
	Lachnospiraceae UCG004	10	MR Egger	1.000 (0.983-1.016)	.956
			Weighted median	1.001 (0.998-1.005)	.408
			IVW	1.002 (1.000-1.005)	**.045**
			Simple mode	1.001 (0.995-1.007)	.694
			Weighted mode	1.001 (0.996-1.007)	.681
	Veillonella	11	MR Egger	1.000 (0.990-1.009)	.927
			Weighted median	1.002 (0.999-1.003)	.191
			IVW	1.002 (1.000-1.004)	**.018**
			Simple mode	1.001 (0.997-1.005)	.564
			Weighted mode	1.001 (0.997-1.005)	.591
Rectal polyp	Allisonella	8	MR Egger	1.000 (0.993-1.008)	.880
			Weighted median	1.001 (1.000-1.002)	.085
			IVW	1.001 (1.000-1.002)	**.048**
			Simple mode	1.001 (1.000-1.003)	.255
			Weighted mode	1.001 (0.999-1.003)	.262
	Christensenellaceae R.7 group	4	MR Egger	1.011 (0.961-1.065)	.705
			Weighted median	0.997 (0.993-1.000)	.078
			IVW	0.997 (0.994-1.000)	**.049**
			Simple mode	0.996 (0.991-1.002)	.288
			Weighted mode	0.996 (0.992-1.001)	.249
	Intestinimonas	11	MR Egger	0.998 (0.985-1.010)	.738
			Weighted median	0.998 (0.996-1.000)	.059
			IVW	0.998 (0.996-1.000)	**.014**
			Simple mode	0.998 (0.994-1.001)	.235
			Weighted mode	0.998 (0.995-1.001)	.213

	Paraprevotella	8	MR Egger	0.996 (0.985-1.007)	.507
			Weighted median	0.998 (0.996-1.000)	.092
			IVW	0.998 (0.997-1.000)	**.028**
			Simple mode	0.997 (0.995-1.000)	.109
Weighted mode			0.998 (0.995-1.001)	.174
Stomach and duodenum polyp	Lachnospiraceae FCS020 group	4	MR Egger	1.066 (0.936-1.213)	.437
			Weighted median	1.002 (1.000-1.004)	.055
			IVW	1.002 (1.000-1.004)	**.032**
			Simple mode	1.003 (1.000-1.005)	.163
			Weighted mode	1.003 (1.000-1.005)	.181
	Intestinibacter	3	MR Egger	1.025 (0.944-1.114)	.657
			Weighted median	1.002 (0.999-1.004)	.163
			IVW	1.002 (1.000-1.004)	**.033**
			Simple mode	1.002 (1.000-1.004)	.318
			Weighted mode	1.002 (0.999-1.005)	.354
	Ruminococcaceae UCG003	4	MR Egger	0.999 (0.932-1.071)	.982
			Weighted median	1.002 (1.000-1.004)	.089
			IVW	1.002 (1.000-1.004)	**.014**
			Simple mode	1.002 (0.999-1.005)	.316
			Weighted mode	1.002 (0.999-1.005)	.291
	Parasutterella	6	MR Egger	0.995 (0.960-1.030)	.776
			Weighted median	1.002 (1.000-1.003)	.054
			IVW	1.001 (1.000-1.003)	**.022**
			Simple mode	1.002 (1.000-1.005)	.142
			Weighted mode	1.002 (1.000-1.005)	.144

IVW, inverse variance weighted; MR, Mendelian randomization; OR, odds ratio; SNP, single-nucleotide polymorphism. bold values represent p < 0.05

**Table 2.** Sensitivity Analyses Between Gut Microbiota and Gastrointestinal Polyps

Outcomes	N snp	MR Method	Heterogeneity		Horizontal Pleiotropy				
			Cochran’s *Q*	*P*	Egger Intercept	SE	*P*	RSS_obs_	*P*
Colon polyp									
*Clostridium innocuum* group	11	IVW	13.876 13.870	.179 .127	−0.000	0.001	.947	16.690	.210
		MR Egger							
Dorea	10	IVW	4.746 3.441	.856 .904	−0.000	0.000	.286	6.037	.855
		MR Egger							
Erysipelotrichaceae UCG003	11	IVW	13.895 13.211	.178 .153	0.000	0.001	.512	16.798	.227
		MR Egger							
Lachnospiraceae UCG004	10	IVW	8.393 8.268	.495 .408	0.000	0.001	.738	10.415	.516
		MR Egger							
Veillonella	11	IVW	4.125 3.846	.942 .921	0.000	0.000	.610	5.046	.951
		MR Egger							
Rectal polyp									
Allisonella	8	IVW	8.041 8.023	.329 .236	0.000	0.001	.912	10.480	.374
		MR Egger							
Christensenellaceae R.7 group	4	IVW	1.251 0.941	.741 .625	−0.001	0.001	.634	2.079	.793
		MR Egger							
Intestinimonas	11	IVW	4.167 4.165	940 .900	0.001	0.000	.970	5.021	.950
		MR Egger							
Paraprevotella	8	IVW	4.900 4.713	.672 .581	0.000	0.001	.680	6.335	.696
		MR Egger							
Stomach and duodenum polyp									
Lachnospiraceae FCS020 group	4	IVW	0.940 0.065	.816 .968	−0.003	0.004	.448	1.666	.837
		MR Egger							
Intestinibacter	3	IVW	0.353 0.054	.838 .816	0.001	0.002	.681	1.362	.756
		MR Egger							
Ruminococcaceae UCG003	4	IVW	2.522 2.513	.471 .285	0.001	0.002	.938	3.126	.589
		MR Egger							
Parasutterella	6	IVW	3.323 3.174	.650 .529	0.001	0.001	.719	5.125	.682
		MR Egger							

The Cochran *Q*-test was used to assess the heterogeneity between the SNP-specific estimates, MR-Egger regression, and MR-PRESSO to test for evidence of pleiotropy.

**Supplementary Table 1.** The detailed information on the Instrumental variables.

https://docs.google.com/spreadsheets/d/1D0AWxztta1we66OxuepYS4bH7LXzQXJA2GoFfkaVHoU/edit?usp=sharing]

**Supplementary Table 2.** Detailed information on the MR results.

[https://docs.google.com/spreadsheets/d/10bmToLQI54jlIcv7ql7kIsQwsK9MRtFYIwkG9jZS0jQ/edit?usp=sharing]

**Supplementary Table 3.** Details of Heterogeneity Analysis.

[https://docs.google.com/spreadsheets/d/1ZcEYg5lCVT2AQfmqhyIi0SB7oas0Ge4y7Rp0qtNSl58/edit?usp=sharing]

**Supplementary Table 4.** Details of Pleiotropy Analysis.

[https://docs.google.com/spreadsheets/d/1jiSw5uLsJ_Vflf43DitbfCtpw4GmJe0M0LwKij0ws7I/edit?usp=sharing]

## Data Availability

The data that support the findings of this study are available on request from the corresponding author.
