# Supplementary material for: Causal Relationship Between Gut Microbiota and Gastrointestinal Polyps: A Mendelian Randomization Study
Source: Turk J Gastroenterol. 2025 Jan 6;36(5):302–11. doi: 10.5152/tjg.2025.24347 (PMC12070428; doi:10.5152/tjg.2025.24347)
Supplement: Supplementary Material [file supplementary_material.pdf]

Supplementary Table 1. The detailed information on the Instrumental variables.

https://docs.google.com/spreadsheets/d/1D0AWxztta1we66OxuepYS4bH7LXzQXJA2GoFfkaVHoU/edit?usp=sharing]

Supplementary Table 2. Detailed information on the MR results.

[https://docs.google.com/spreadsheets/d/10bmToLQI54jllcv7ql7kIsQwsK9MRtFYIwkG9jZS0jQ/edit?usp=sharing]

Supplementary Table 3. Details of Heterogeneity Analysis.

[https://docs.google.com/spreadsheets/d/1ZcEYg5lCVT2AQfmqhyli0SB7oas0Ge4y7Rp0qtNSl58/edit?usp=sharing]

Supplementary Table 4. Details of Pleiotropy Analysis.

[https://docs.google.com/spreadsheets/d/1jiSw5uLsJ_Vflf43DitbfCtpw4GmJe0M0LwKij0ws7I/edit?usp=sharing]

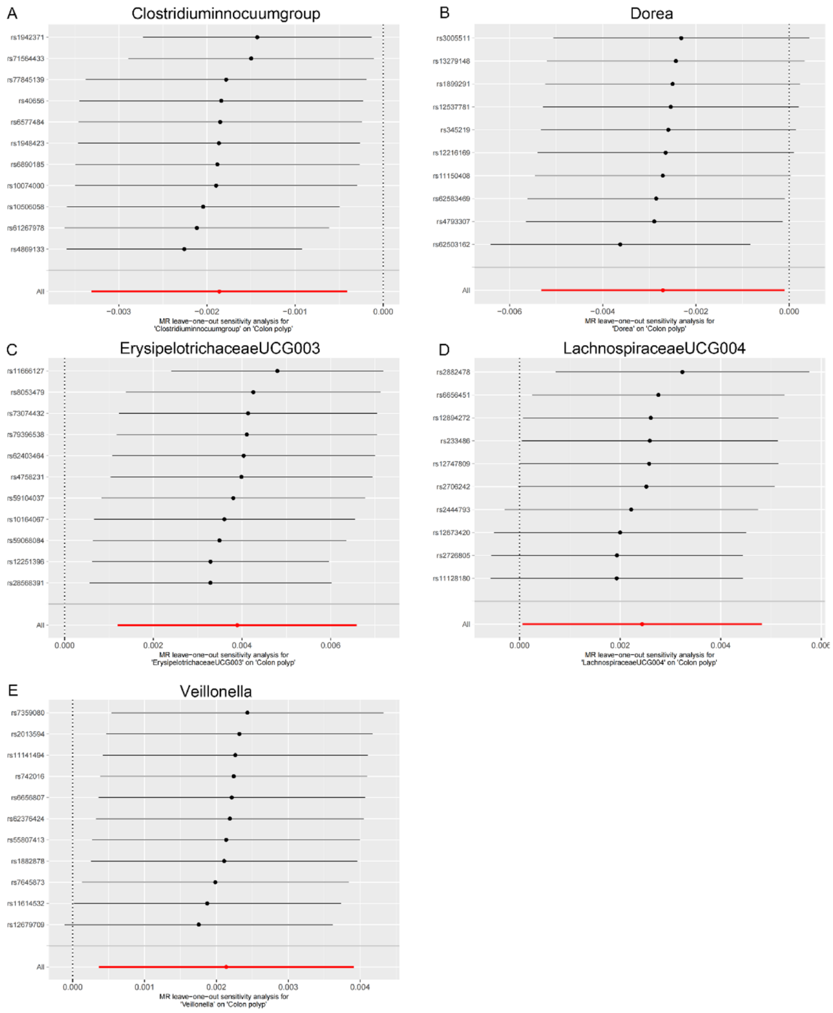

Supplementary Figure 1. Leave-one-out sensitivity analysis based on the IVW model for the association between gut microbiota and colon polyp. (A) *Clostridium innocuum* group; (B) Dorea; (C) Erysipelotrichaceae UCG003; (D) Lachnospiraceae UCG004; (E) Veillonella. The red horizontal line represents the overall estimate, while the black horizontal line represents the estimate after removing a single variant. Abbreviations: SNP, single-nucleotide polymorphism; IVW, Inverse variance weighted.

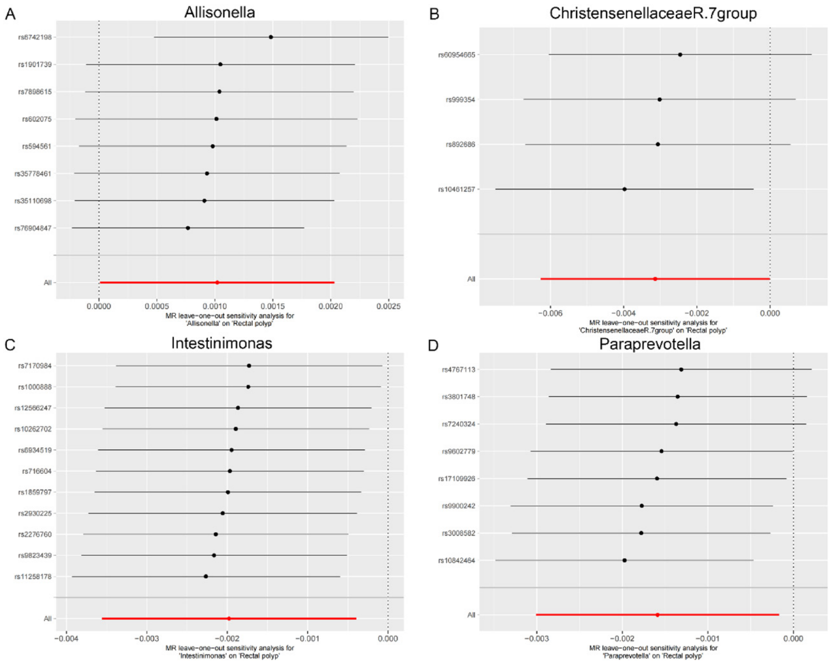

Supplementary Figure 2. Leave-one-out sensitivity analysis based on the IVW model for the association between gut microbiota and rectal polyp. (A) Allisonella; (B) Christensenellaceae R.7 group; (C) Intestinimonas; (D) Paraprevotella. The red horizontal line represents the overall estimate, while the black horizontal line represents the estimate after removing a single variant. Abbreviations: SNP, single-nucleotide polymorphism; IVW, Inverse variance weighted.

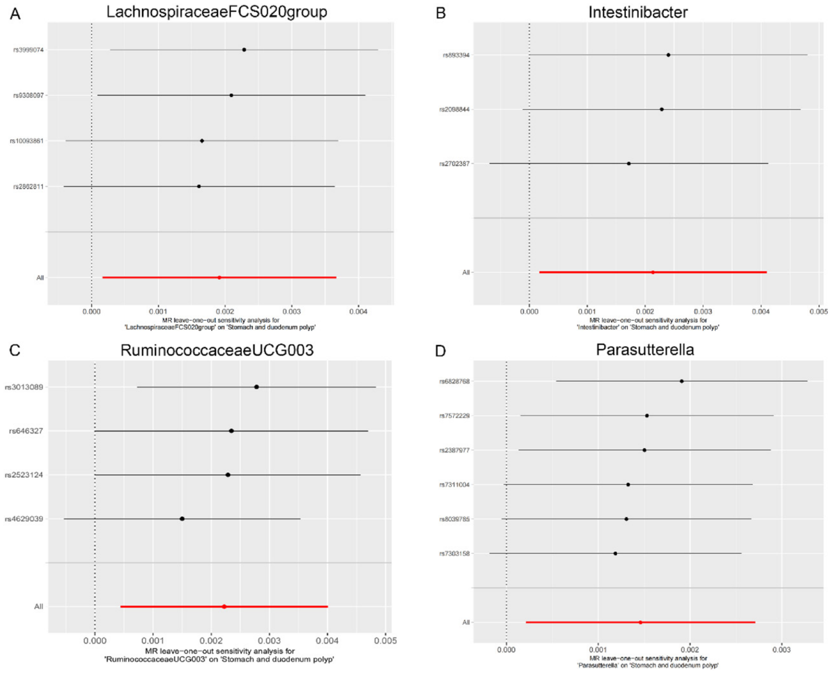

Supplementary Figure 3. Leave-one-out sensitivity analysis based on the IVW model for the association between gut microbiota and stomach and duodenum polyp. (A) Lachnospiraceae FCS020 group; (B) Intestinibacter; (C) Ruminococcaceae UCG003; (D) Parasutterella. The red horizontal line represents the overall estimate, while the black horizontal line represents the estimate after removing a single variant. Abbreviations: SNP, single-nucleotide polymorphism; IVW, Inverse variance weighted.

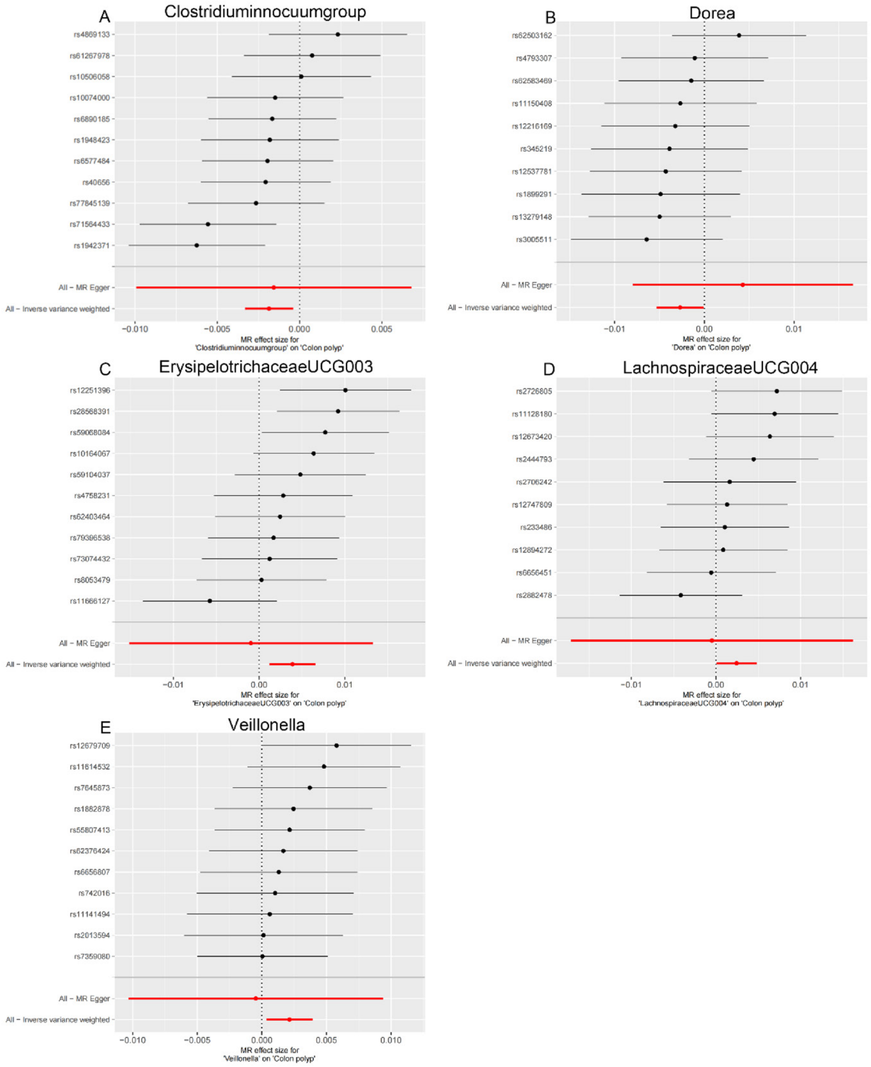

Supplementary Figure 4. Forest plot for the association between Gut microbiota on colon polyp. (A) *Clostridium innocuum* group; (B) Dorea; (C) Erysipelotrichaceae UCG003; (D) Lachnospiraceae UCG004; (E) Veillonella. Inverse Variance Weighting (IVW) and MR Egger methods were used to detect the heterogeneity of SNP.

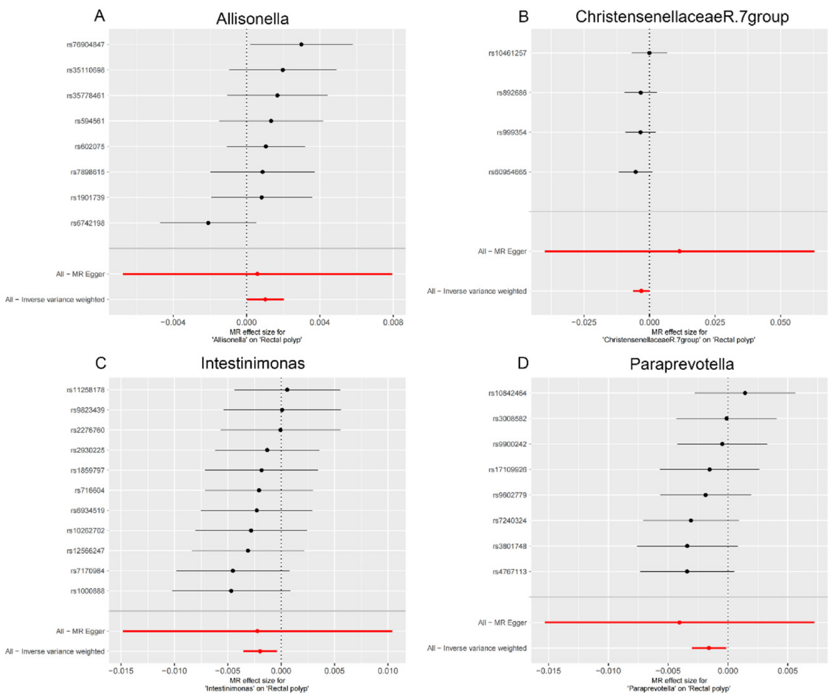

Supplementary Figure 5. Forest plot for the association between Gut microbiota on rectal polyp. (A) Allisonella; (B) Christensenellaceae R.7 group; (C) Intestinimonas; (D) Paraprevotella. Inverse Variance Weighting (IVW) and MR Egger methods were used to detect the heterogeneity of SNP.

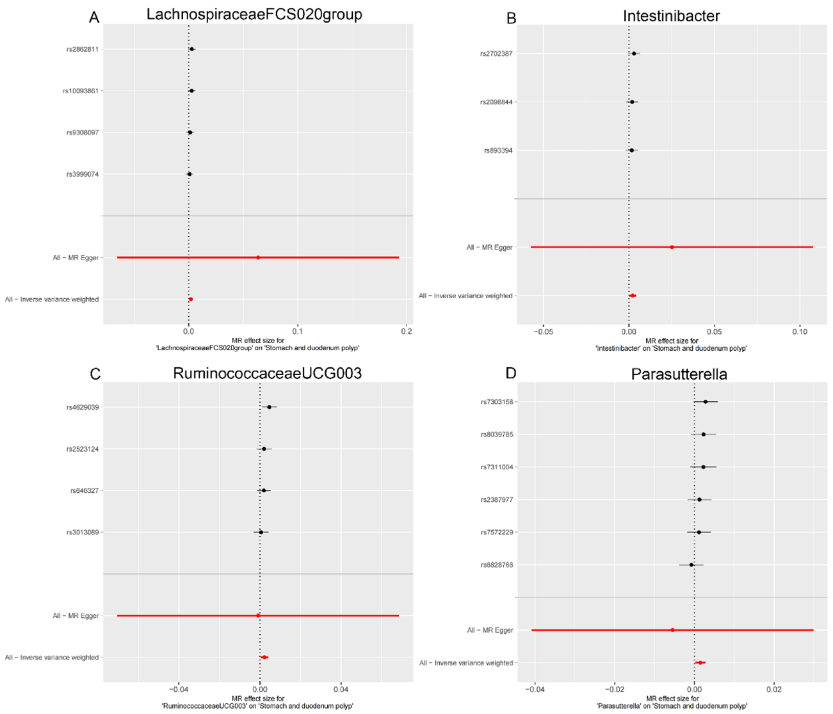

Supplementary Figure 6. Forest plot for the association between Gut microbiota on stomach and duodenum polyp. (A) Lachnospiraceae FCS020 group; (B) Intestinibacter; (C) Ruminococcaceae UCG003; (D) Parasutterella. Inverse Variance Weighting (IVW) and MR Egger methods were used to detect the heterogeneity of SNP.

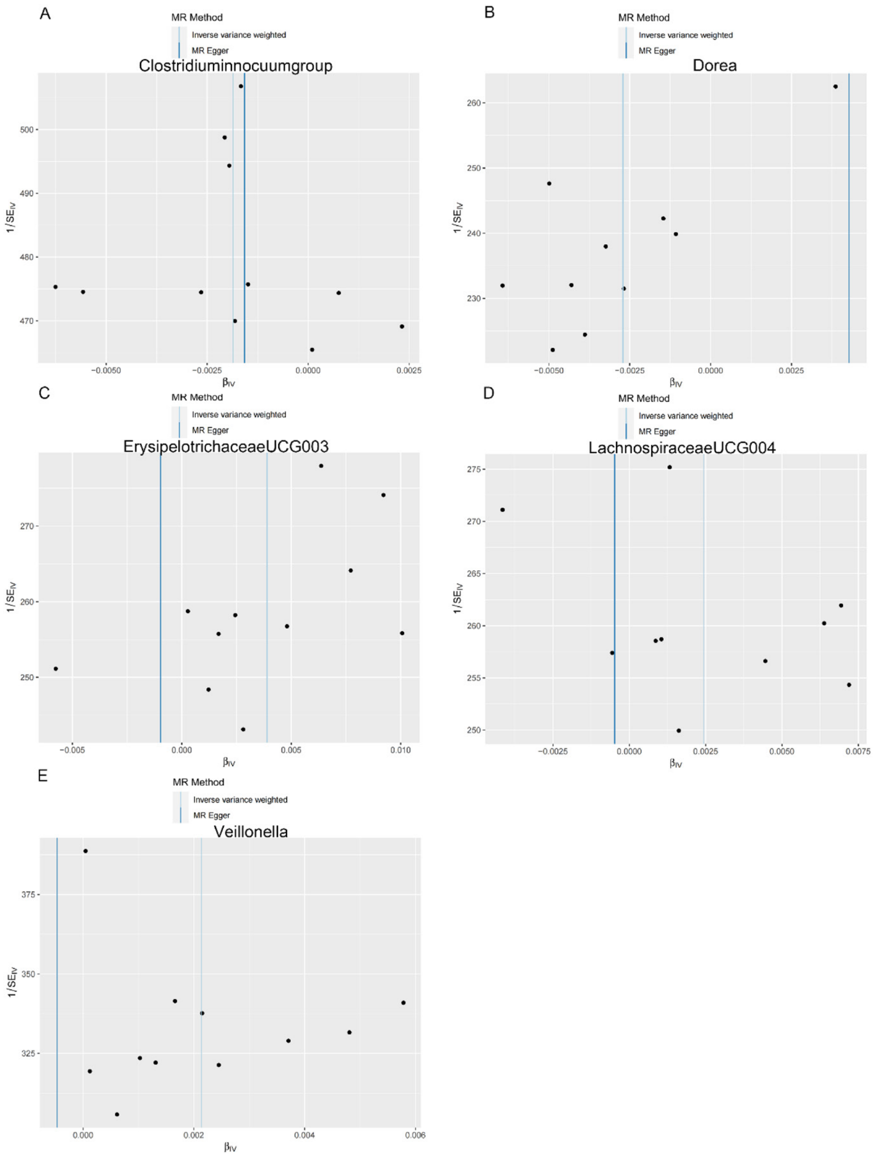

Supplementary Figure 7. Funnel plots for MR analyses of the causal effect of Gut microbiota on colon polyp. (A) *Clostridium innocuum* group; (B) Dorea; (C) Erysipelotrichaceae UCG003; (D) Lachnospiraceae UCG004; (E) Veillonella. Inverse Variance Weighting (IVW) and MR Egger methods were used to detect the heterogeneity of SNP.

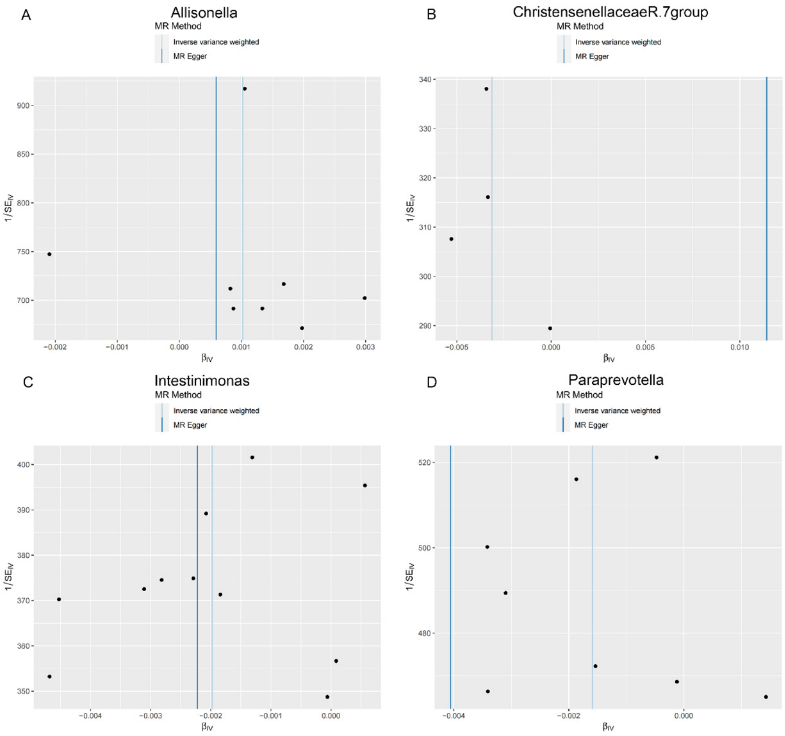

Supplementary Figure 8. Funnel plots for MR analyses of the causal effect of Gut microbiota on rectal polyp. (A) Allisonella; (B) Christensenellaceae R.7 group; (C) Intestinimonas; (D) Paraprevotella. Inverse Variance Weighting (IVW) and MR Egger methods were used to detect the heterogeneity of SNP.

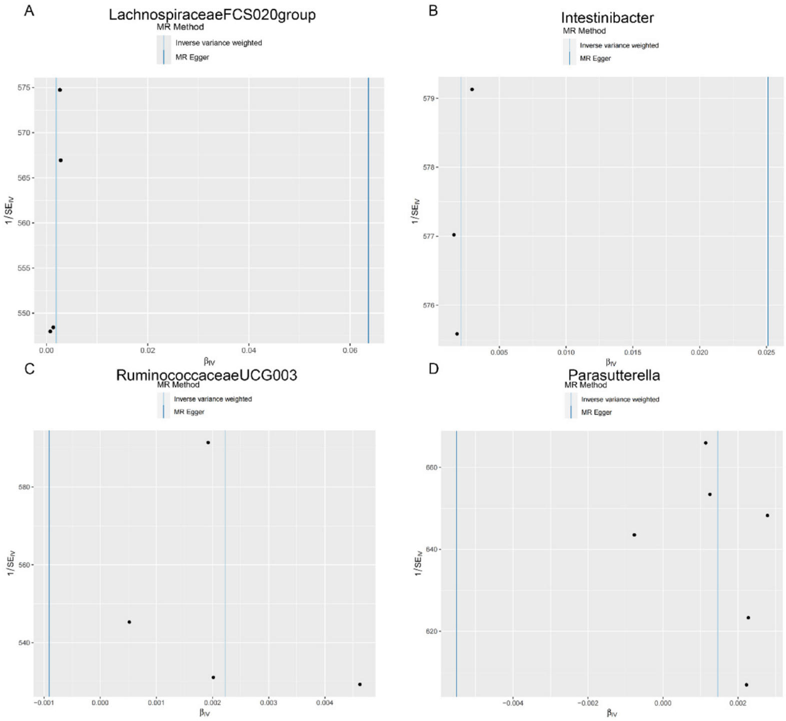

Supplementary Figure 9. Funnel plots for MR analyses of the causal effect of Gut microbiota on stomach and duodenum polyp. (A) Lachnospiraceae FCS020 group; (B) Intestinibacter; (C) Ruminococcaceae UCG003; (D) Parasutterella. Inverse Variance Weighting (IVW) and MR Egger methods were used to detect the heterogeneity of SNP.
